# The role of counselling in tuberculosis diagnostic evaluation and contact tracing: scoping review and stakeholder consultation of knowledge and research gaps

**DOI:** 10.1186/s12889-022-12556-8

**Published:** 2022-01-28

**Authors:** Isabel Foster, Amanda Sullivan, Goodman Makanda, Ingrid Schoeman, Phumeza Tisile, Helene-Mari van der Westhuizen, Grant Theron, Ruvandhi R. Nathavitharana

**Affiliations:** 1 International Development and Research Centre, Ottawa, Canada; 2 TB Proof, Cape Town, South Africa; 3 Division of Infectious Diseases, Beth Israel Deaconess Medical Center/Harvard Medical School, Boston, MA USA; 4 Department of Primary Care Health Sciences, University of Oxford, Oxford, UK; 5 DSI-NRF Centre of Excellence for Biomedical Tuberculosis Research, South African Medical Research Council Centre for Tuberculosis Research, Division of Molecular Biology and Human Genetics, Stellenbosch University, Cape Town, South Africa

## Abstract

**Background:**

Tuberculosis (TB) care cascade analyses show large gaps at early stages, including care-seeking and diagnostic evaluation, where promising interventions to decrease attrition are urgently needed. Person-centered care is prioritized in the World Health Organization’s End TB strategy; yet little is known about how it is delivered and can be optimized. Recommendations for counselling, a core component of person-centered care, are largely limited to its role in improving TB treatment adherence. The role of counselling to close key diagnostic gaps in the care cascade is poorly understood.

**Methods:**

We conducted a scoping review to identify evidence on the use of counselling at TB diagnosis, for both people with presumptive TB and index patients to promote patient retention and contact tracing. Using search terms for TB, diagnosis and counselling, we systematically searched PubMed, EMBASE and Web of Science. Two independent reviewers screened all abstracts, full-texts, extracted data and conducted a quality assessment. We used thematic analysis to identify key themes.

**Results:**

After screening 1785 articles, we extracted data from 15 studies and determined that the major themes best corresponded to the following gaps in the TB care cascade: care-seeking, pre-diagnosis, and pre-treatment. Studies were conducted across varied settings including pharmacies, primary health centres, and clinics, primarily in high TB incidence countries. No study directly evaluated the impact of counselling on outcomes such as treatment initiation or retention in care. Included studies suggested counselling may play an important role in improving the uptake of diagnostic testing and contact tracing. Barriers to counselling included time and personnel requirements. Stakeholder consultation emphasized the importance of high-quality counselling as a core tenet of TB care.

**Conclusion:**

Data on the impact of counselling to improve TB case detection are absent from the literature. The shift towards person-centred care for TB presents an opportunity to incorporate counselling during earlier stages of the TB care cascade; however, evidence-based approaches are needed. Implementation research is needed to operationalize and evaluate counselling to strengthen high-quality TB care delivery.

**Supplementary Information:**

The online version contains supplementary material available at 10.1186/s12889-022-12556-8.

## Background

Tuberculosis (TB) remains remains a major cause of death due to an infectious disease worldwide [1]. In 2020, an estimated 10 million people became sick with TB, of whom only 6 million were notified to national TB programs, demonstrating an increase in the TB diagnostic gap as a result of the COVID-19 pandemic [1]. Patients being evaluated for TB are required to navigate long, often circuitous [2], pathways of care which, when compounded with low quality user experience, leads to substantial disengagement from TB care services [3]. A re-design of TB care service delivery is critical to curbing transmission in high burden regions; expanding coverage and utilization alone is insufficient [4, 5].

Cascade of care analyses based on national program data from high burden countries show that TB care delivery is greatly impeded by three ‘gaps’ [6, 7]; (1) the care seeking gap – the period during which patients or their contacts may be infectious but have not been evaluated; (2) the pre-diagnostic gap – whereby patients have been tested but not yet diagnosed; and (3) the pre-treatment gap – patients have received a positive TB diagnosis but have not been registered for treatment. In addition to efforts to strengthen active case finding to diagnose index patients with TB, contact tracing is a high yield strategy for identifying additional people with TB [8]. There is an urgent need to identify promising interventions that decrease patient attrition during early stages of the cascade [9].

The WHO’s End TB strategy for 2015–2035 places patient-centred care at the forefront [10]. Patient-centred care emphasizes that support programs must be ‘responsive to patients’ educational, emotional and material needs’ [10] but the precise definition of services that constitute people-centred care remains ambiguous [11]. To enable the provision of high quality care more broadly at the health systems level, proper counselling and health education has been described as an essential element of evidence-based care [12]. While the WHO strongly recommends counselling as a component of comprehensive patient-centred TB care, this is only discussed in the context of improving treatment adherence rather than at earlier stages in the care cascade [13].

Currently, little is known about the impact of counselling offered to people being evaluated for TB to improve diagnostic yield or to people diagnosed with TB to improve retention in care and facilitate contact investigation. This precludes the formulation of precise questions on the effectiveness of such interventions that could be assessed in a systematic review. There has also been limited engagement with key stakeholders, particularly from affected communities, to understand their perspectives on how the user experience of TB care services can be improved. The purpose of our scoping literature review and stakeholder consultation was to examine existing data on counselling services offered to people being evaluated for TB at the point of diagnosis or people diagnosed with TB to facilitate contact tracing, and identify practice and research gaps.

## Methods

This scoping review follows the Arksey and O’Malley framework [14] and the Joanna Briggs Institute 2015 recommendations [15]. The study involved five phases: (1) identification of the research questions; (2) determination of relevant studies; (3) study selection; (4) data charting, quality assessment, and stakeholder consultations; and (5) collation and summary of findings. We followed the preferred reporting items for systematic and meta-analyses extension for scoping reviews (PRISMA-ScR) checklist (see Additional file 1) to report our study findings.

### Phase 1: refining of study scope and research questions

Our overall research question sought to understand how counselling is delivered to patients being evaluated for TB (irrespective of presentation with or without symptoms) and patients who are diagnosed with any form of TB, and whether counselling increases the uptake and yield of diagnosis and contact tracing compared to care delivery approaches that do not include counselling. We obtained input from stakeholders with lived experiences of TB as well as clinician researchers to formulate this question and to assist with identifying relevant research and operational examples.

### Phase 2: determination of relevant studies

As recommended by Arksey and O’Malley [14] a two-step search strategy was used. An initial search of PubMed was undertaken followed by an analysis of the text words contained in the title and abstract of relevant articles, and of the index MESH terms used to describe the articles. Based on the initial search, we refined our search terms for TB, diagnosis, contact tracing and counselling using identified keywords and index terms (Additional file 2) and undertook a revised search across the following databases (PubMed, EMBASE, Web of Science). All searches were conducted by a medical librarian and results imported into Covidence [16].

### Phase 3: study selection

This scoping review considered all studies that included counselling offered to people being evaluated for or diagnosed with any form of TB (including drug resistant TB), or contacts of index patients diagnosed with TB. Studies published in English were considered for inclusion in this review and no restriction was placed on publication year. Studies were not restricted based on any contextual settings including geographic location, country income status or TB incidence rate. We included studies that assessed interventions that included counselling alongside other support services, and studies that assessed counselling alone. We did not restrict by study type and included any relevant quantitative, qualitative or mixed methods studies, and reviews (systematic or other).

Articles were screened for relevance based on the title and abstract. This was followed by a full-text review of articles identified from initial screening. At each stage of the screening process, two independent reviewers screened and extracted data (IF and AS). Disagreements were resolved through discussion with a third reviewer (RRN).

### Phase 4: data charting, quality appraisal and stakeholder consultation

Data was extracted from the included papers using a pre-piloted charting table (Additional file 3). The charting table incorporated data on study authors and year of publication, country, health system context, study design, target population, sample size, analysis methods, primary study findings, impact of counselling, details about how and when counselling was delivered and if this was specifically an intervention that was studied. Two reviewers (IF and AS) extracted data independently. Any disagreements that arose between the reviewers were resolved through discussion with a third reviewer (RRN).

The quality rating of all studies included was graded by two reviewers (IF and AS) using a modified version of the 2018 mixed-method quality appraisal tool (MMAT) [17]. Upon answering screening questions for each study design (qualitative, quantitative, and non-randomized control), a total percentage was calculated. Studies which scored less than 50% were of low quality, between 51 and 70% average quality and above 70% high quality.

To build on and contextualise our findings, we undertook a stakeholder consultation with TB survivors, advocates and clinician researchers. Using a focus group format (conducted in English which was spoken by all stakeholders) to facilitate knowledge transfer and group discussion, we shared preliminary findings of the review and asked them to discuss issues related to counselling based on their expertise and experiences and to identify emerging priorities for research on counselling at the point of TB diagnosis and contact tracing as part of efforts to advance person-centered TB care. IF and RRN took field notes during the focus groups, listened to the audio recordings of the focus group and undertook independent coding in Word to categorize data from the focus groups inductively into themes. Themes were then compared to develop a coding scheme which was applied to the transcripts [14, 18, 19].

### Phase 5: collation and summary of findings

**Fig. 1:**
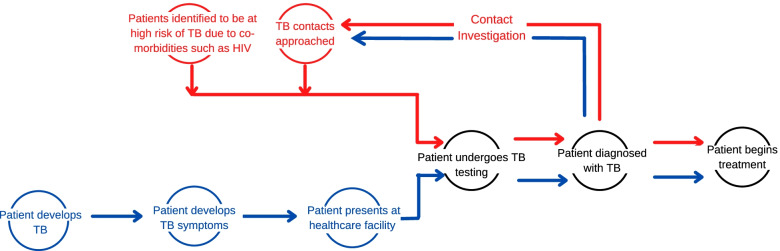
The provider initiated (red) and patient initiated (blue) pathways of care are long, with many opportunities for patients to be lost to follow up at each stage. We deliberately ended the cascade steps (black) in this figure at treatment initiation, since this review is focused on evaluating the potential role of counselling to decrease earlier cascade gaps

We explored the role of counselling at different diagnostic stages of the TB care cascade which patients may initiate through one of two pathways: patient-initiated and provider-initiated (Fig. 1). Three reviewers (IF, AS and RRN) used the principles of thematic analysis [14, 20], to identify common themes that emerged. Results were organized by thematic categories. Findings related to the implementation and impact (measured or hypothesized) of counselling are presented narratively.

## Results

**Fig. 2:**
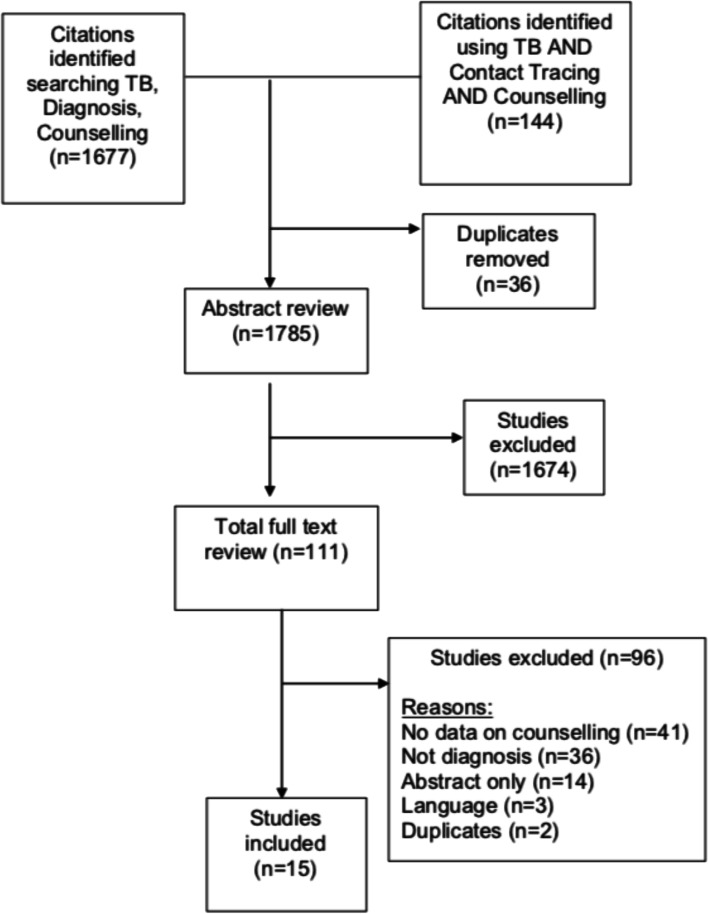
PRISMA flow diagram demonstrating the process of identification and selection for articles to be included in the scoping review

The database search identified 1821 articles, of which 36 were duplicates. Of the remaining 1785 articles, 1674 were excluded. Of the 111 full texts assessed for eligibility, 96 were excluded, primarily due to not containing data on counselling, TB diagnosis or contact tracing. Overall, 15 studies were included (Fig. 2).

### Characteristics of the included studies

The characteristics of included studies and findings related to counselling are in Table 1. Studies took place in a variety of health contexts, including community pharmacies, ward-based outreach teams, TB centres, chest clinics, households, tertiary care centres primary health centres, and out-patient clinics. Most studies (87%, 13/15) were in countries with a high incidence of TB [1]. Most studies reported data on interventions that included counselling at the time of diagnostic evaluation for active TB (87%, 13/15), aside from two studies reporting data on counselling at the time of diagnostic evaluation for latent TB. The majority of studies (*n* = 13) focused on drug-sensitive TB (DS-TB), aside from two studies that focused on people with drug-resistant TB (DR-TB). Counselling was used to improve a variety of outcomes such as completion of diagnostic testing and improving yield of sputum collection (Table 1). All studies were published between 1999 and 2020, with the majority (73%, 11/15) published between 2015 and 2020. Most studies used cross-sectional research designs (80%, 12/15); one used a case-study design and two used operational research designs to assess the program implementation. No study used a comparator group to test the validity of findings.

**Table 1:** Characteristics of included studies that reported data on interventions that included counselling at the time of diagnostic evaluation for active TB

Author, Year	Country, context, study population	Study Aim and Design	How was counselling used or mentioned in the study?	Study findings related to counselling	Potential barriers to counselling	Quality of Study [17]
Care Seeking Gap						
Ullah, et al. 2020 [21]	Pakistan Community pharmacies 3025 presumptive TB cases	Implementation study to assess the feasibility and yield of community pharmacy-based TB case detection.	Community pharmacies provided referral slips to patients with presumptive TB (based on symptoms or purchase of anti-TB medication). For patients who re-presented to the pharmacy but had not followed up on referral, pharmacy staff provided counselling.	1901 patients in receipt of a referral actually visited GP clinics (referral uptake = 63%) and 547 cases were diagnosed with TB, that is, a positive referral outcome of 18%. Every fifth referral among presumptive cases presenting and counseled at pharmacies was diagnosed with TB at GP clinics.	Lack of incentives for pharmacists	60%
Putra et al. 2018, [22]	Indonesia Public health center 365 patients with diabetes mellitus (DM) type II being evaluated for TB	Nested cross-sectional qualitative study with structured questionnaires to identify factors associated with participation in pulmonary TB screening.	This study was nested within a TB diabetes screening study for which participants received counselling delivered by HCPs for pulmonary TB screening using chest x-ray.	Multivariate analysis showed that patients who received good support from their HCP, in the form of counselling was associated with participation in pulmonary TB screening [adjusted prevalence ratio = 1.35, 95% CI (1.06–1.70)]. Despite counselling, patient knowledge and attitudes related to TB and diabetes as co-morbidities were poor, highlighting the importance of high quality counselling.	Training HCPs in the delivery of high quality counselling. Need to engage family members during the counselling process.	60%
Belgaumkar et al. 2018 [23]	India Tertiary referral hospital 80 adults with smear positive pulmonary TB, 49 child contacts, and 25 health care providers (HCPs)	Cross-sectional study using semi-structured questionnaires and health record review to evaluate screening and isoniazid preventive therapy (IPT) provision among child contacts.	This was a programmatic evaluation (rather than an intervention study) of contact tracing referral and IPT uptake. Per program guidelines, index patients should receive counselling regarding contact screening and IPT.	Index cases with no counselling by HCPs (*p* < 0.001, adjusted OR [OR] 19.7) were less likely to have their child contact screened. 56/80 (70%) index patients were not counseled about TB risk and screening in child contacts. 39/56 (70%) said they were willing for screening and preventive therapy for child contacts if recommended. 19/25 (76%) HCPs said they routinely recommended index patients to bring their child contacts for screening. 20/24 (86%) of index patients who received advice about TB screening adhered.	The majority of index patients were reluctant to bring child contacts for screening as they did not have power to decide (i.e. were not the parent) (94%) and they did not think that the child would get TB (60%)	60%
Kumar et al. 2013 [24]	United Kingdom TB clinics 1 index patient, 15 contacts	Case study describing a TB outbreak within a UK family where proven widespread transmission occurred but initial contact tracing yield was low.	Close contacts who screened negative for both LTBI and active TB were advised to be aware of symptoms and signs of disease affecting themselves and others.	Re-screening of contacts (who had received initial counselling) after one contact with LTBI developed active TB disease identified TB disease in 6/19 and LTBI in 8/19.	Buy-in from both the medical team is needed, since the educational component of counselling can be time consuming.	20%
Furin et al. 2020, [25]	South Africa Households 8 patients with drug-resistant TB and 8 supporters	Retrospective, Cross-sectional qualitative using in-depth interviews to describe the meaning of ‘people centred care’.	The semi-structured interview guide included questions about challenges with drug-resistant TB diagnosis and care, and sources of support during care.	Few patients had support before formal diagnosis and this was usually from family members/spouses (almost always females). Nurses were identified as the focal points for person-centered care but needed further training to provide counselling.	Multiple care providers at different facilities. Co-ordination and communication between them sub-optimal.	100%
Khan et al. 2006 [26]	Pakistan Out-patient clinics 170 current and former TB patients (112 public sector, 58 private sector)	Cross sectional study using questionnaires to assess knowledge, attitudes and misconception about TB.	Questionnaire asked whether patients had received counselling about preventing TB transmission.	81/170 (48%) patients reported receiving no counselling by their physicians about how to prevent the spread of infection.	Inadequate knowledge of and misconceptions about TB on the part of general practitioners. TB-related stigma.	40%
Pre-diagnostic gap						
Kivihya-Ndugga, et al. 2007 [27]	Kenya Chest Clinic 1469 patients with presumptive TB	Nested cross sectional cohort study to evaluate completion of the TB diagnostic process after counselling and to identify factors that impact adherence to recommended diagnostic process.	During evaluation of patients with presumptive TB, trained nurses provided counselling with a focus on obtaining three quality sputum specimens for evaluation	95% of the patients with presumptive TB who received counselling from trained nurses provided 3 sputum samples. There was no comparison group but this is higher than reported in other published data.	Counselling took 0.5 h/per patient. Lack of staff capacity to undertake counselling.	60%
Bonsu et al. 2017, [28]	Ghana TB clinics 35 clinic staff	Qualitative study using in-depth interviews to highlight healthcare professionals’ perspectives on patient satisfaction.	The interview guide was unstructured but one of the major themes highlighted was counselling and education.	Respondents frequently mentioned the need for patient counselling/education as core to satisfying TB patients, with three specific components: provision of TB related knowledge including transmission prevention, helping patients to cope with the diagnosis including stigma, and to provide education on appropriate techniques for providing sputum.	Long waiting times. TB-related stigma.	70%
Kirsch et al. 1999 [29]	United States Tertiary care center 630 presumptive TB cases	Implementation study to assess the feasibility and effectiveness of an emergency department (ED)-based TB screening and counselling program conducted in cooperation with the local public health department.	ED patients identified as being high-risk for having latent TB were counselled about TB and post-counselling assessment evaluated the patient’s understanding of purified protein derivative (PPD) testing and rates of follow up for PPD reading.	873 patients were counselled, 630 were eligible for screening, and 374 (59.4%) consented to purified protein derivative PPD testing. Of the 203 (54.1%) who returned, 32 (15.8%) were PPD-positive. Initial counselling took an average of 28 min per patient. Enrollment with postcounselling testing, reeducation, and PPD placement took an additional 70 min. Although it was not independently evaluated, counselling was highlighted as an important aspect of the study due to its influence over patients and their contacts with regard to seeking screening.	Training on counselling needed for program managers/all clinical staff. High staff turnover. Long waiting times. Nurses unable to prescribe treatment.	50%
Pre-treatment Gap						
Islam et al. 2015 [30]	Bangladesh DOTS Center, Chest Clinic and Tertiary care center 4974 referred cases, 234 TB patients from the referred cases and 30 healthcare providers	Quantitative study using structured questionnaires and record review to identify the gaps in the referral system, including the pre-treatment gap.	Patients were asked about ability to follow instructions given during counselling. HCPs were interviewed regarding their knowledge about counselling and the process of referral.	Ability to follow instructions during counselling was significantly associated with identification of DOTS centres by patients who remained in the referral system. Only 40% of health workers interviewed had the experience of referring TB patients to the DOTS centres through proper counselling.	Ensuring that patients can follow instructions provided during counselling. Many patients are diagnosed by private providers who do not provide effective counselling.	70%
McNally et al. 2019 [31]	Peru Healthcare centers or households 15 current or former MDR patients and 11 HCPs	Qualitative study using semi-structured interviews to examine patient perceptions, experiences and views on positive and negative factors that impact outcomes.	Counselling was not explicitly evaluated or mentioned but themes included patient knowledge and education.	Patients mention knowledge gaps and those with poor knowledge saw their education as the responsibility of HCPs. HCPs acknowledged the importance of quality patient education and mentioned the importance of the method of delivery and source of the information.	An initial distrust of medical advice. Inadequate clinical infrastructure. TB-related stigma.	60%
Mwansa-Kambafwile et al. 2020 [32]	South Africa Ward-based outreach teams and TB programming 9 program managers	Qualitative study using in-depth interviews to explore reasons for TB initial loss to follow up from the perspectives of TB and primary care program managers.	Interview guide included questions about TB related communication and reasons for loss to follow up.	Lack of counselling for TB (in comparison to HIV) mentioned as a reason for loss to follow up.	Staff reluctance to work in ‘TB room’. Frequent staff rotations. Staff shortages.	50%
Colvin et al. 2019 [33]	The Philippines Health facilities 560 patients and 435 TB service providers	Cross-sectional quantitative study using questionnaire to identify and address gaps in the quality of TB services.	The study utilized a quality of TB services assessment that included questions about whether counselling was provided or received and included a series of items related to interpersonal counselling and communication (IPCC) skills were analyzed.	The analysis shows that providers consistently reported having covered basic TB information more often than patients reported receiving the information during counselling. While 77% of providers reported that they discussed duration of TB treatment, only 33% of clients reported knowing how long treatment would last. When clients reported lower levels of IPCC, their recall of key topics covered in counselling was lower.	Training of health care providers to improve communication and counselling skills	60%
Mntlangula et al. 2017 [34]	South Africa Primary health center 87 nurses	Cross sectional quantitative study using self- administered questionnaires to assess the knowledge, attitude and beliefs of nurses about behavioral counselling for HIV and AIDS, sexually transmitted diseases and TB (HAST).	Counselling was the focus of the questionnaires, which were based on the Health Belief Model.	Although the majority of nurses were in favor of the counselling behavior for HAST, 54 (62%), (95% CI: 50.0, 71.0) believed that poverty stricken patients only need treatment since they cannot do anything to improve their health. Some nurses had a negative attitude towards counselling behaviour for HAST, for example whether there was a benefit for patients with alcohol use disorders or for patients with good adherence.	Insufficient time to counsel patients properly and insufficient space. Negative attitudes of HCPs regarding counselling may lead to counselling not being undertaken.	50%
Ayakaka et al., 2017 [35]	Uganda Out-patient clinics and one general hospital 61 health care workers, 21 lay health workers (LHW), and 400 household contacts of newly diagnosed TB patients	Cross-sectional qualitative study using focus group discussions and interviews to identifying barriers to and facilitators of TB contact investigation in Kampala, Uganda.	Counselling was discussed as part of several themes that emerged from the data.	HCPs mentioned that tasks like TB education and counselling were often viewed as being of a low priority and thus ignored. Patients mentioned that counselling provided by LHWs motivated them to initiate treatment promptly.	Insufficient personnel at TB unit. Lack of dedicated space for TB care. Fear of contracting TB among clinic staff. TB-related stigma Distrust of clinic-staff among contacts.	80%

### Quality assessment

All included articles underwent methodological quality appraisal using MMAT [17]. The majority (73%) scored 60% or below (range 20–100%), indicating low quality, often due to unaccounted confounders in non-randomized studies, high risk of non-response bias in quantitative studies, and interpretation of results not sufficiently substantiated by data in qualitative studies. Due to the limited data available, no studies were excluded based on quality.

### Primary review findings

**Fig. 3:**
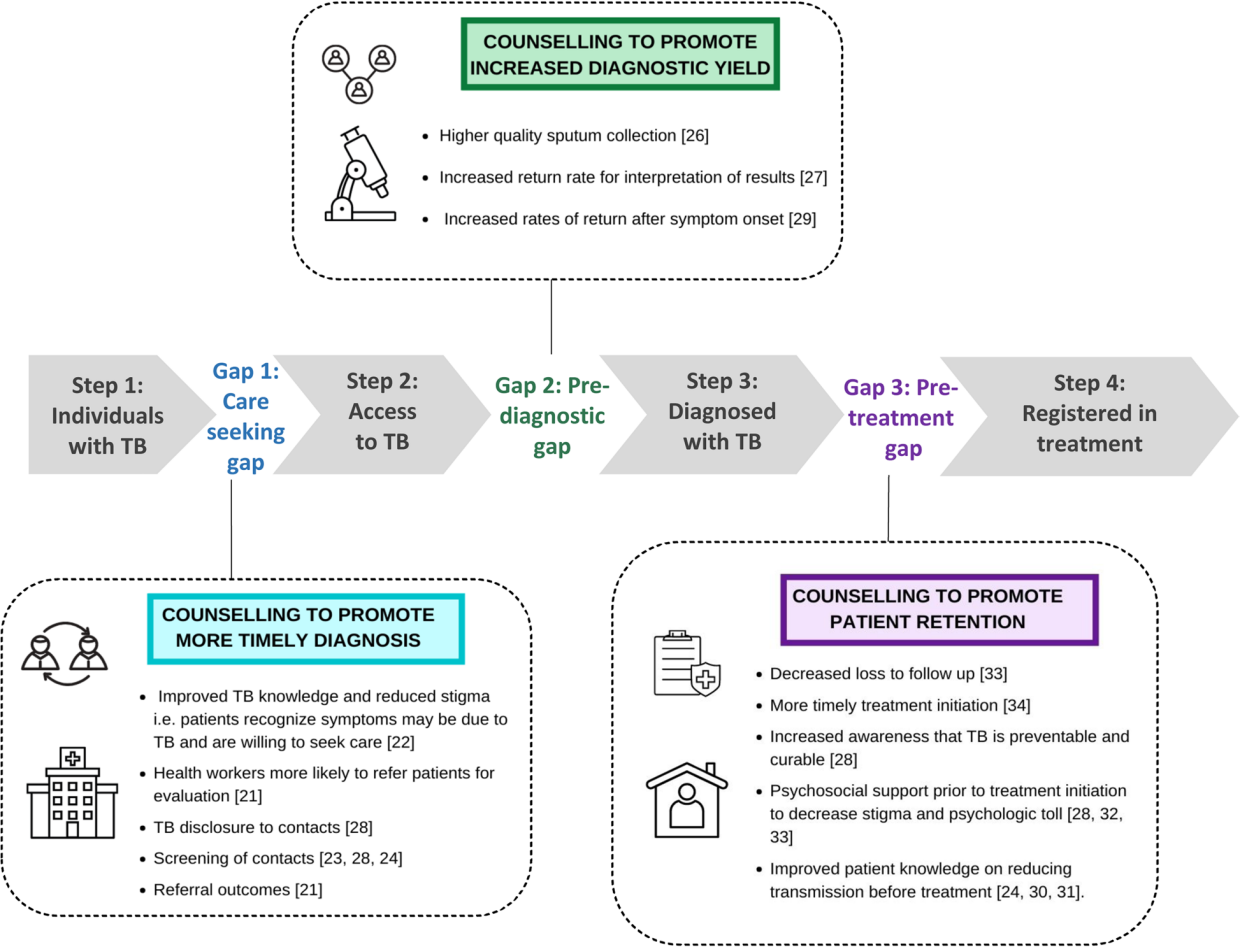
A high proportion of patients disengage from care during three key gaps that occur before initiating treatment [6, 7]. The boxes demonstrate specific examples for which counselling has the potential to decrease each of these gaps by increasing timely diagnosis, improving community and individual knowledge, increasing diagnostic yield and improving patient retention in care

We considered the role of counselling provided to people being evaluated for or diagnosed with any form of TB, including index patients and contacts. No studies directly evaluated the impact of counselling on TB care cascade outcomes. Based on thematic analysis, we determined that the major themes of the scoping review best corresponded to each gap of the diagnostic care cascade: care-seeking, pre-diagnosis, pre-treatment (see Fig. 3). We identified barriers to counselling across different cascade stages for these respective pathways.

#### Care-seeking gap

##### Patient initiated pathway: referral of symptomatic patients for TB evaluation

When community pharmacists were trained in symptomatic screening and counselling to promote referral to general practice clinics for TB evaluation in rural Pakistan, 1901 patients who received a referral from one of these trained community pharmacists visited GP clinics (referral uptake = 63%, *n* = 3025). Of the referred, 547 were diagnosed with TB, resulting in a positive referral outcome of 18% [21].

##### Provider initiated pathway: screening of high-risk groups

High risk groups present an important target population for screening interventions. A study in Indonesia in which patients with diabetes presenting to public health centres who received counselling from HCPs about pulmonary TB screening with chest x-rays reported that support provided by HCPs, in the form of information provided through counselling, was associated with increased participation in pulmonary TB screening [adjusted prevalence ratio = 1.35, 95% CI (1.06–1.70)] [22]. However, patient knowledge and attitudes related to TB-DM comorbidity (84 and 61% respectively, *n* = 365) were poor, demonstrating a gap in the delivery of quality counselling that can improve knowledge and facilitate decision-making.

##### Provider initiated pathway: contact investigation

Counselling at the point of index patient diagnosis can facilitate the screening of pediatric contacts who may have been exposed to infection. In a cohort of 80 patients with smear positive pulmonary TB in India, index patients who reported not receiving counselling were less likely to have their children screened for TB (*P* = 0.001, adjusted OR [aOR] 19.7) [23]. Another study found that disclosure is sometimes avoided to mitigate stress, whereby nearly 40% of patients (*n* = 170) did not reveal their disease status to friends and family for fear of stigma [26].

#### Pre-diagnostic gap

Counselling may facilitate completion of TB diagnostic evaluation. In a nested cross-sectional cohort study in Kenya, after receiving counselling from a research nurse, 95% of participants completed diagnosis, measured by provision of 3 sputum samples. The validity of the results are difficult to determine given that there was no control group [27]. A qualitative study in Ghana reported that HCPs thought that counselling could ensure that patients were more likely to produce quality sputum and thus help to obtain reliable results in a shorter time [28].

Counselling may facilitate the yield of TB diagnostic evaluation. Counselling of patient groups with TB risk factors presenting to an emergency department in the United States was associated with a relatively high proportion of patients (54%, *n* = 203) who returned to have their tuberculin skin test readings. Counselling was however noted to be the most time consuming aspect of the programme [29].

##### Drug-resistant TB

South African patients with DR-TB in a qualitative study reported feeling physically sick (but not knowing the underlying cause of their symptoms) and visiting at least two healthcare facilities prior to their DR-TB diagnosis [25].

##### Provider initiated pathway: contact investigation

An analysis of a TB outbreak in the United Kingdom reported high rates of return for evaluation by contacts who had received counselling at the time of their initial screening and re-presented when they later developed symptoms consistent with TB. The authors suggest that educational counselling using empowering rather than coercive language helped contacts to engage or reengage with the health system [24].

#### Pre-treatment gap

Several studies suggested that effective counselling during diagnostic evaluation could educate patients with presumptive or confirmed TB about TB transmission to decrease community spread [26, 33, 34]. In one study, participants with TB in Pakistan were found to have profoundly poor knowledge about TB transmission, whereby 57% (*n* = 96) of participants considered separating dishes as the most commonly used method for preventing spread of disease [26]. Just under 50% of patients in this study reportedly did report not receiving any counselling from their health care provider about how to prevent transmission [26].

Although some South African study participants with MDR-TB qualitative study reported that they were told their diagnoses in a sympathetic manner, others reported that either minimal counselling was provided or nurses evoked fear in the people being evaluated for TB [25].

‘…They did not explain very clearly what was happening. I had the paper with me and then they called me to check the paper. They then said come here and put a mask on me. They told me to sit outside and wait there. They did not treat me well [25]’

Several studies reported that counselling could potentially ease the transition from diagnosis to treatment initiation [30, 32]. A study in Bangladesh demonstrated that patients’ ability to follow instructions during counselling was significantly associated with identification of DOTS centres by patients who remained in the referral system. However, only 12/30 (40%) of health workers had experience of referring patients to Direct Observational Therapy (DOTS) centres for treatment initiation through counselling [30]. In a qualitative study among HCPs in South Africa, the lack of patient counselling was expressed as a primary reason for initial loss to follow up prior to treatment initiation [32].

‘Patients are not counselled when they are tested …you know like in HIV. So the patients do not know the importance of starting treatment soon. So they just go home. And are given a date when to come back and this is where the gap is. They normally don’t come back on their own [32]’.

A cross-sectional study of HCPs and contacts in Uganda reported that patients mentioned counselling by lay health workers helped them to better understand the link between treatment initiation and cure, motivating them to seek care more promptly [35].

‘If they have explained very well like the community worker told us if you have the sickness and you start the medication early, you get better faster… I wouldn’t wait… I would want to get healed and wouldn’t want to be sick for a long time. I would run to the health unit for treatment because she explained [that] very well to me… [35]..

A study from the Philippines that analysed questionnaires from both patients recently diagnosed with TB and HCPs showed that while 77% (*n* = 330) of providers reported discussing the duration of treatment, only 33% of patients (*n* = 428) reported knowing the anticipated duration of treatment [33]. Importantly, when patients reported lower scores on an assessment of HCP interpersonal counselling and communication skills, patient recall of key information covered during counselling was lower, highlighting the need for institutionalized training of HCPs in effective counselling techniques [33].

Several studies highlighted the threat that TB poses to patients’ mental health and wellbeing. Qualitative studies from South Africa and Ghana reported few patients received emotional support from family members after diagnosis [25] and patients struggle to cope with their TB diagnosis [28]. Counselling post diagnosis by ancillary staff including peer support networks was suggested as a strategy to help patients address fears about upcoming treatment [25]. HCPs in Ghana reported counselling patients about TB being curable and preventable could help them accept their diagnosis and address the mental and emotional burden that a TB diagnosis often generates [28].

‘An individual diagnosed with TB may feel condemned to death. There must be counselling to work on their mental and emotional state and let them know that anybody in the world can be infected with TB. We also let them know that TB is curable and preventable so we let them go through this, to understand gradually and accept their present state of condition [28].’

##### Drug-resistant TB

TB-related stigma was discussed as a known barrier to care within a cohort of South African patients with DR-TB, who mentioned that messages about DR-TB were often delivered with the goal of evoking fear including terms such as ‘most dangerous [form of TB]’ [25]. Study participants also reported that disclosing one’s TB status to close contacts can be associated with high personal costs, including feelings of guilt and stress and that little support to manage these was provided by the healthcare system.

A qualitative study in Peru that interviewed both HCPs and MDR-TB patients, found that effective education is an important factor that enables patients to come to terms with their diagnosis. An MDR-TB diagnosis affected patients and their families significantly. Support, both within families and the healthcare setting, was important to enable patients to overcome the impact of an MDR-TB diagnosis [31].

#### Barriers to counselling

**Fig. 4:**
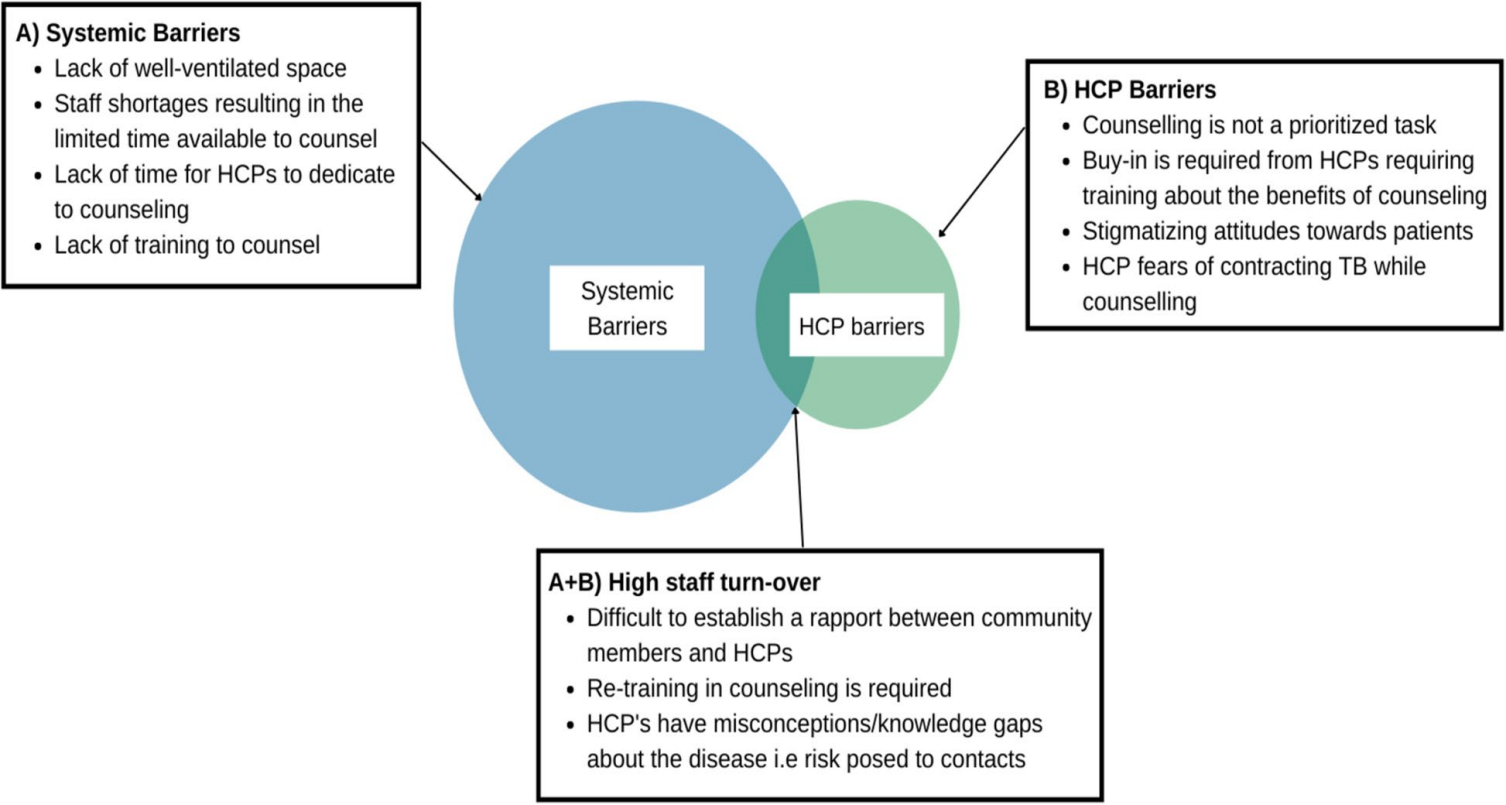
Systemic and health care provider related barriers impact the delivery and likely success of counselling interventions. This review found more systemic barriers than HCP related ones, suggesting a need for national TB control programs to place greater priority on policy, guidelines, and resources to facilitate the implementation of counselling

A combination of system and HCP-related barriers to counselling such as high staff turnover [32], lack of training [23] and shortage of appropriate spaces [31] were frequently reported amongst the included studies (Fig. 4). HCPs and patients in Peru reported the lack of rooms available for private conversations and adequately ventilated space [31]. Patients often see multiple care providers across different facilities, hindering continuity of care [25]. High staff turnover makes it difficult for TB patients as they often have to reacquaint themselves to new staff who may have variable attitudes toward both the disease and counselling [32, 34]. Several studies reported that improving the delivery of effective counselling was contingent on improving the knowledge, attitude and willingness of HCPs [27, 29, 35]. Training of staff also presents a barrier to counselling. Studies showed that staff are not familiar with protocols for counselling TB patients [29, 35], including discussion of the risk posed to their contacts [23].

Furthermore, the necessary workforce is often not in place or existing health providers have competing commitments that compromise the implementation of effective counselling [27, 29, 35]. The buy-in required from medical staff presents a barrier to counselling, due to the time it consumes amidst busy schedules [24]. In a study in South Africa, 35% (*n* = 87) of nurses reported not having sufficient time to counsel patients [34]. HCPs in Uganda mentioned that tasks such as TB education and counselling were considered to be of low priority and thus were ignored [35].

HCPs’ misconceptions around the disease also hinders commitment to providing counselling. Stigma towards patients was reported in a cohort of HCPs in South Africa in which 62% of the sample (95% CI: 50.0, 71.0 *N* = 87) believed that ‘poverty stricken patients just need treatment as they cannot do anything to improve their health’ [34]. Fear of infection of TB may also affect the care delivered by HCPs. The same study reported that 19.5% (95% CI: 12.0,29.0) of nurses were concerned about contracting TB while counselling patients [34].

### Stakeholder consultation

While stakeholder opinions closely reflected the findings of the review, they also yielded additional recommendations. Our stakeholders reiterated that based on their experiences in South Africa, counselling during diagnostic evaluation is not standard practice across different TB types, such as differences in the approach to care for drug-sensitive TB versus DR-TB, with the latter group more likely to receive counselling, and care settings. They perceived that TB counselling tends to be prioritized for outpatients but was also important for inpatients. They also recommended that patients testing negative for TB should receive counselling to understand under what circumstances they should return for diagnostic evaluation and emphasized the importance of community based advocacy about TB testing to encourage community members to present for evaluation when they have TB related symptoms. The stakeholders drew comparisons to HIV and mentioned the need for counselling to cover specific information beyond the need to take treatment, which should include guidance on the likelihood and duration of infectiousness, modes of transmission and the need for contact tracing, and address mental health and wellbeing.

Stakeholders mentioned the heavy reliance on informal support networks, NGOs or the discretion of individual HCPs. As such, many TB patients fall through gaps and receive little support or guidance. Stakeholders emphasized the importance of language, both in terms of providing counselling in a language that patients understand and the need to avoid stigmatizing language. Recommended research priorities included implementation research to understand how, when and where counselling can best be performed. The counselling agenda should be directly informed by people affected by TB and perspectives from community leaders and traditional healers. To operationalize counselling as a standard practice during diagnosis, stakeholders recommended national TB programs should strive for a holistic, person-centered care that moves beyond only measuring quantifiable outcomes (such as adherence and cure rates) to evaluating outcomes that are meaningful to people affected by TB.

## Discussion

No studies included in this review directly evaluated the impact of counselling as an intervention to improve TB diagnostic care cascade outcomes. Studies included in this review and stakeholder consultation suggest that that counselling can address three key hurdles that need to be overcome to decrease care seeking and diagnostic gaps in the TB cascade of care. Firstly, counselling provided at the initial point of diagnostic evaluation may improve diagnostic yield and promote retention of patients who enter the TB cascade through both provider and patient-initiated pathways of care. This is important in the context of 50% of people with TB being lost to follow up between presenting for TB evaluation at a health centre and initiating TB treatment [6, 7, 11]. Since these patients have already presented for evaluation, this represents an easy to utilize opportunity that is currently often missed [36] to engage them by providing individualized, holistic, empowering and respectful care [37]. Secondly, studies showed that TB remains a heavily stigmatized disease. As such, disclosing TB status, particularly with regard to DR-TB, presented a significant personal and societal cost to patients. Counselling is one potential component of support that could help to overcome this barrier however, this should be provided in combination with other interventions that target the drivers and manifestations of stigma across all socio-ecological levels [38]. Thirdly, counselling could improve awareness and reduce stigma at the community level, leading to patients presenting for evaluation earlier and being empowered to decrease community transmission. Moreover, counselling can play an important role in improving patient experience, which may also improve mental wellbeing. Low-quality care is associated with approximately 50% of TB deaths [4] and poor user experience contributes to this statistic [4]. TB survivors, including those who participated in our stakeholder consultation, have identified the need for counselling to understand diagnostic testing options and to explain the implications of diagnosis as part of efforts to improve the quality of TB care [3, 39]. Interventions that improve user experience should be prioritized as part of the WHO’s shift toward person-centred care.

Although studies included in this review highlighted the potential role that counselling can have before testing, we found significant heterogeneity across studies in terms of their approach to implementing counselling, including reporting of how and by whom it was provided. We note that studies found that HCPs often do not provide counselling [30], and that HCPs recalled providing counselling more often than patients reported receiving it [33], highlighting important discrepancies between patient and provider perspectives on the quality of care delivery. Studies emphasized the barriers that stigmatization of TB presents, highlighting a practice and research gap to understand whether and how counselling could improve TB related knowledge and attitudes [25, 32, 35]. We also note that studies showed limited support (not restricted to the lack of counselling alone) is currently offered from local health systems to augment the supplemental efforts of family members [23, 25, 31] or volunteer support (as underscored in our stakeholder consultation).

Our study findings have implications for practice. Guidelines from the WHO and International Standards for TB care (ISTC) only refer to counselling in the context of patients on TB treatment or for those being evaluated for HIV. Although we chose not to include unpublished data as part of our review, as part of our stakeholder consultation we identified programmatic and policy documents pertaining to counselling at the time of diagnosis. The objectives of training curriculum developed by the global non-profit PATH for the Ukraine Tuberculosis Control Partnership Project [40] were aligned with the findings of this review. The curriculum identified pre-test counselling as an ideal point to introduce patient support, develop trust and to provide patients with basic information to protect household members before treatment initiation [40]. Other identified documents focused on counselling for HIV and/or limited TB related counselling to treatment adherence. More recently, the International Union Against Tuberculosis and TB Alert released a guide ‘Psychological Counselling and Treatment Adherence Support for People with Tuberculosis’. The ‘Barriers in Diagnosis’ section provides some guidance on providing psychosocial support for people being screened or tested for TB [41]. While there were few studies on DR-TB and none that compared counselling for DS-TB to DR-TB, our stakeholder consultation highlighted that services for counselling and follow up, although primarily in the context of treatment rather than diagnosis, are often more robust for DR-TB. We did not restrict study inclusion based on country income status and note that although there are likely to be more resources for counselling in richer countries with a lower TB incidence, we were not able to find evidence of standardized diagnostic counselling practices. It is likely that gaps in the TB care cascade vary by country and practice settings; thus emphasizing the importance of local TB care cascade evaluation to guide the development of interventions such as counselling [9]. The limited number of heterogeneous studies across different settings do not enable comparison of resource or cost implications for incorporating diagnostic counselling into practice.

Strengths of our scoping review included conducting a systematic search of the literature, that was not limited by country setting or date, in consultation with a research librarian, followed by screening, extraction and evidence grading by at least two independent reviewers. By including only peer-reviewed articles, ensuring that our eligibility criteria were rigorously applied, and performing methodological quality appraisal at the individual study using the MMAT tool, we have endeavored for this scoping review to uphold similar quality standards to a systematic review and maximise our ability to evaluate the potential impact of counselling on TB care outcomes. Through the stakeholder analysis that we undertook as part of this review, we were able to incorporate key perspectives from people directly affected by TB. Limitations include only examining studies in English.

Our review demonstrates the need for well-conducted operational and implementation research to evaluate and optimize the role of counselling within TB care, which was also emphasized during our stakeholder consultation (Table 2.). Quantifying the research gap is an important first step that can facilitate intervention design. This raises an important question to be addressed in future research; how often is person-centered care (including counselling) provided to patients? Multi-countries studies using standardized patients have highlighted how diagnosis of TB is a key hurdle to overcome for effective care [42]. These studies, however, use safety and efficacy indicators to measure the “correct” management of presumptive patients. Indicators to assess the degree of user experience should also be used in such types of studies (i.e. provider’s attitude, communication, explanations received etc. [4]). Mixed methods approaches that include qualitative data collection from in-depth interviews and focus groups with key stakeholders can help to identify contextual barriers and facilitators. We suggest that quasi-experimental or randomized controlled trials should be used to evaluate the impact of counselling on TB care outcomes. Future studies should compare the effectiveness of counselling provided by different HCPs and examine the acceptability and feasibility of scaling up different approaches, as well as the differential needs for people with DS-TB compared to DR-TB. Barriers to improving TB care through counselling, such as TB stigma [11], must be addressed as part of any strategy to improve TB care.

**Table 2:** Recommendations for research studies: a) to quantify whether and how counselling is being delivered, b) to understand patient and HCP needs that could be fulfilled by counselling and c) to evaluate the impact of counselling on patient-important TB care outcomes

Type of research	Examples:
Operational or Implementation research To quantify the extent to which diagnostic counselling is currently offered and evaluate delivery strategies, which could be incorporated as part of programmatic efforts.	Standardized patient studies that include indicators regarding diagnostic counselling (for patient support, explaining procedures, disclosure counselling etc.) that quantify whether, how and by whom counselling is being delivered as well as the quality of counselling.
	Studies to test different approaches to the delivery of counselling, including assessment of by whom the counselling intervention should be implemented e.g. health workers (and type) versus peer navigators.
	Pre-post studies using tools such as Knowledge, Attitudes, and Practices surveys to assess gaps in patient and health worker understanding and practice.
Qualitative studies Interviews and/or focus groups with patients with TB, caregivers of people with TB and health workers to determine which topics should be addressed by counselling.	Evaluating the optimal approach to training HWs on counselling and interpersonal communication skills.
	Use of explanatory frameworks to understand drivers of loss to follow up and potential roles for counselling including different types of behavioural change techniques.
Intervention studies Rigorously designed and conducted studies to test the impact of diagnostic counselling with a comparator group.	Quasi-experimental or randomised controlled trials to evaluate the impact of diagnostic counselling on TB cascade of care outcomes.
	Translating insights from counselling research conducted for other stigmatized illnesses (i.e. HIV, mental illness) to TB and developing integrated care models.

## Conclusions

Our scoping review is the first to comprehensively explore the potential role of counselling during TB diagnostic evaluation. Although there are no studies demonstrating the impact of counselling to decrease gaps related to diagnosis and screening in the TB care cascade, data from both quantitative and qualitative studies suggest it may play an important role and merits further evaluation. The poor overall quality of evidence identified highlights the need for high quality implementation research. As global and national guidelines attempt to operationalize person-centred TB care, counselling at the early stages of the cascade of care must be considered, not only to increase diagnostic yield and patient retention, but as a means to introduce holistic care from the outset, that fulfils the right to high-quality care, as endorsed by TB survivors. While this review is focused on TB, we hope that the insights on counselling as a core-tenet of person-centred care may also be of relevance to other diseases, particularly other stigmatized infections.

## Supplementary Information

**Additional file 1.**

**Additional file 2.**

**Additional file 3.**

## Data Availability

Interview guide used for the stakeholder consultation for this is available from the corresponding author on request.
