# Supplementary material for: The role of counselling in tuberculosis diagnostic evaluation and contact tracing: scoping review and stakeholder consultation of knowledge and research gaps
Source: BMC Public Health. 2022 Jan 28;22:190. doi: 10.1186/s12889-022-12556-8 (PMC8795719; doi:10.1186/s12889-022-12556-8)
Supplement: Supplementary file 3 — Additional file 3. [file 12889_2022_12556_MOESM3_ESM.docx]

**Search strategy for scoping review:**

PubMed Search Strategies:

TB AND Diagnosis AND Counseling (536 Results)

("tuberculosis"[mesh] OR "tuberculosis"[tiab] OR "tuberculosis, multidrug-resistant"[mesh] OR "multidrug-resistant tuberculosis"[tiab] OR "mycobacterium tuberculosis"[mesh] OR "mycobacterium tuberculosis"[tiab] OR "TB"[tiab]) AND ("diagnosis"[mesh] OR "diagnosis"[tiab] OR "diagnostic maging"[mesh] OR "diagnostic imaging"[tiab] OR "screening"[tiab] OR "testing"[tiab] OR "diagnostic evaluation"[tiab]) AND ("counseling"[mesh] OR "counseling"[tiab] OR "counselling"[tiab])

TB AND Contact Tracing AND Counseling (28 Results)

("tuberculosis"[mesh] OR "tuberculosis"[tiab] OR "tuberculosis, multidrug-resistant"[mesh] OR "multidrug-resistant tuberculosis"[tiab] OR "mycobacterium tuberculosis"[mesh] OR "mycobacterium tuberculosis"[tiab] OR "TB"[tiab]) AND ("contact tracing"[mesh] OR "contact tracing"[tiab] OR "contacts"[tiab] OR "tracing contacts"[tiab]) AND ("counseling"[mesh] OR "counseling"[tiab] OR "counselling"[tiab])

EMBASE Search: TB AND Diagnosis AND Counseling

12. #9 AND #10 AND #11 (939 results)

11. #5 OR #6 OR #7 OR #8

10. #1 OR #2 OR #3 OR #4’

9. ‘counseling’/exp OR ‘counseling’

8. ‘testing’/exp OR ‘testing’

7. ‘screening’/exp OR ‘screening’

6. ‘diagnostic imaging’/exp OR ‘diagnostic imaging

5. ‘diagnosis’/exp OR ‘diagnosis’

4. ‘extensively drug resistant tuberculosis’/exp OR ‘extensively drug resistant tuberculosis’

3. ‘mycobacterium tuberculosis’/exp OR ‘mycobacterium tuberculosis’

2. ‘multidrug resistant tuberculosis’/exp OR ‘multidrug resistant tuberculosis’

1. ‘tuberculosis’/exp OR ‘tuberculosis’

EMBASE Search: TB AND Contact Tracing AND Counseling

8. #5 AND #6 AND #7 (10 results)

7. #1 OR #2 OR #3 OR #4

6. ‘counseling’/exp OR ‘counseling’

5. ‘contact examination’/exp OR ‘cpntact examination’

4. ‘extensively drug resistant tuberculosis’/exp OR ‘extensively drug resistant tuberculosis’

3. ‘mycobacterium tuberculosis’/exp OR ‘mycobacterium tuberculosis’

2. ‘multidrug resistant tuberculosis’/exp OR ‘multidrug resistant tuberculosis’

1. ‘tuberculosis’/exp OR ‘tuberculosis’

Web of Science Search:

TBDiagnosisCounseling:

4. #3 AND #2 AND #1 (930 results)

3. TS=(counseling OR counselling)

2. TS=(diagnosis OR diagnostic imaging OR screening OR testing OR diagnostic evaluation)

1. TS=(tuberculosis OR mycobacterium tuberculosis OR multidrug resistant tuberculosis OR extensively drug resistant tuberculosis OR TB)

TBContactTracingCounseling

6. #5 AND #3 AND #1 (101 results)

5. TS=(contact tracing OR contact examination OR contacts OR tracing contacts)

4. #3 AND #2 AND #1

3. TS=(counseling OR counselling)

2. TS=(diagnosis OR diagnostic imaging OR screening OR testing OR diagnostic evaluation)

1. TS=(tuberculosis OR mycobacterium tuberculosis OR multidrug resistant tuberculosis OR extensively drug resistant tuberculosis OR TB)

Cochrane Library Search:

Search Name: TB AND Diagnosis AND Counseling (237 results)

#1 MeSH descriptor: [Tuberculosis] explode all trees 1951

#2 tuberculosis 7069

#3 MeSH descriptor: [Mycobacterium tuberculosis] explode all trees 309

#4 mycobacterium tuberculosis 1075

#5 multidrug resistant tuberculosis 407

#6 extensively drug resistant tuberculosis 85

#7 TB 6130

#8 MeSH descriptor: [Diagnosis] explode all trees 329063

#9 diagnosis 149445

#10 MeSH descriptor: [Diagnostic Imaging] explode all trees 47009

#11 diagnostic imaging 31963

#12 screening 57497

#13 testing 56343

#14 diagnostic evaluation 15132

#15 MeSH descriptor: [Counseling] explode all trees 5335

#16 counseling 23254

#17 #1 OR #2 OR #3 OR #4 OR #5 OR #6 OR #7 10980

#18 #8 OR #9 OR #10 OR #11 OR #12 OR #13 OR #14 496162

#19 #15 OR #16 23877

#20 #17 AND #18 AND #19 237

Search Name: TB AND Contact Tracing AND Counseling (54 results)

#1 MeSH descriptor: [Tuberculosis] explode all trees 1951

#2 tuberculosis 7069

#3 MeSH descriptor: [Mycobacterium tuberculosis] explode all trees 309

#4 mycobacterium tuberculosis 1075

#5 multidrug resistant tuberculosis 407

#6 extensively drug resistant tuberculosis 85

#7 TB 6130

#8 MeSH descriptor: [Contact Tracing] explode all trees 75

#9 contact tracing 221

#10 contact examination 4191

#11 contacts 4146

#12 tracing contacts 63

#13 MeSH descriptor: [Counseling] explode all trees 5335

#14 counseling 23254

#15 #1 OR #2 OR #3 OR #4 OR #5 OR #6 OR #7 10980

#16 #8 OR #9 OR #10 OR #11 OR #12 8138

#17 #13 OR #14 23877

#18 #15 AND #16 AND #17 54

LILACS Search:

1. Tuberculosis OR TB (Words)
2. Diagnosis OR diagnostic imaging (Words)
3. Counseling OR counselling (Words)
4. #1 AND #2 AND #3 (2 results)
5. Tuberculosis OR TB (Words)
6. Contact tracing OR contacts (Words)
7. Counseling OR counselling (Words)
8. #1 AND #2 AND #3 (0 results)

The JBI Database of Systematic Reviews and Implementation Reports was searched; the search yielded zero (0) results.
